# Response to Commentary: Accounting for diverse transposable element landscapes is key to developing and evaluating accurate de novo annotation strategies

**DOI:** 10.1186/s13059-023-03119-0

**Published:** 2024-01-02

**Authors:** Shujun Ou, Ning Jiang, Candice N. Hirsch, Matthew B. Hufford

**Affiliations:** 1 Department of Molecular Genetics, Ohio State University, Columbus, OH 43210 USA; 2 Department of Horticulture, Michigan State University, East Lansing, MI 48824 USA; 3 Department of Agronomy and Plant Genetics, University of Minnesota, Saint Paul, MN 55108 USA; 4 Department of Ecology, Evolution, and Organismal Biology, Iowa State University, Ames, IA 50011 USA

**Keywords:** Transposabel Element, Annotation, Benchmarking

EDTA is an evolving tool for transposable element (TE) annotation that is under continual development. We are a group of plant biologists; hence, EDTA 1.0 was developed and benchmarked for use primarily in plant genomes, in which non-long terminal repeat (LTR) retrotransposons contribute only a small fraction of TE content. A major limitation that was discussed in the original paper describing EDTA for broad application was the lack of reliable tools at that time (May 2019) for structural annotation of non-LTR retrotransposons, including long interspersed nuclear elements (LINEs) and short interspersed nuclear element (SINEs), and the over inclusiveness of the search engine for Helitrons [1]. Since its original release, we have continued to make improvements that address concerns raised by users. Below, we detail new benchmarking that was done in response to the specific concerns raised in the commentary by Gozashti and Hoekstra [2], ongoing improvements to EDTA, and best practices for using the software when applying to species with variable TE landscapes.

EDTA first begins with structural annotation of intact transposable elements using specialized annotation programs (e.g., TIR-Learner to annotate terminal inverted repeat (TIR) elements and LTR_retriever to annotate LTR retrotransposons). EDTA then builds a filtered, non-redundant library of intact TE sequences from structurally annotated elements to perform homology-based annotation of non-intact TE sequences in the genome. At this stage, a user is able to provide a reference library to augment the library generated from the EDTA-identified, structurally intact elements. If a user is annotating a species with a TE landscape that is not reflected in the structural annotation tools currently included in EDTA (e.g., LINEs and SINEs), EDTA will not perform optimally when run using default settings. In this situation, it is helpful to provide an additional reference library to EDTA. This recommendation was made in the original manuscript describing the EDTA program [1] and is detailed on the current EDTA GitHub repository (https://github.com/oushujun/EDTA).

Unfortunately, previous knowledge of species-specific TE sequences is not always available to a researcher. However, general databases of repeat sequences exist that can be provided to EDTA. For example, Repbase [3] is a well-curated reference database of repeat sequences potentially useful for EDTA annotation of eukaryotes. To test for performance differences using a general database of sequences, we benchmarked EDTA using the full set of non-LTR sequences in the Repbase database (v24.03) as an added reference library with abundant non-LTR sequences from 109 different species (e.g., zebrafish, mouse, fly, rice, and maize). Benchmarking was done for seven species that included both animal and plant species that have a range of TE landscapes and resources including: chicken, fly, maize, mouse, rice, zebrafish, and zebra finch. We observed that species with good representation in the database (e.g., zebrafish, mouse) showed improved sensitivity and classification accuracy for non-LTR retrotransposons. However, for species that are not well represented in the database (e.g., chicken, zebra finch), the performance improvement was marginal.

To improve annotation of non-LTR retrotransposons in species that are not well-represented in Repbase, we next tested supplementing EDTA annotation with RepeatModeler2 [4] identified non-LTR sequences. This benchmarking was done using the same seven species as above. The incorporation of RepeatModeler2 non-LTR results into EDTA resulted in an acceptable sensitivity for non-LTR retrotransposon annotation comparable to running only RepeatModeler2 in both animals and plants (Table 1). The slight decrease in sensitivity is balanced by the distinct advantages of EDTA in generating annotations of structurally intact elements along with homology-based annotation of non-intact elements. Additionally, the benchmarking demonstrated high sensitivity and specificity for other TE types when running EDTA supplemented with non-LTR retrotransposon sequences from RepeatModeler2, which improves upon the utility of RepeatModeler2 (Table 1). With the incorporation of RepeatModeler2 results, EDTA becomes more generalized to both plant and animal genomes with a diversity of TE landscapes. Still, there is room to improve. For example, the SINE annotation in both EDTA and RepeatModeler2 is marginal and would benefit from the incorporation of specialized, high-quality de novo annotation tools.

**Table 1:** Benchmarking whole-genome TE annotations on plant and animal genomes including chicken, fly, maize, mouse, rice, zebrafish, and zebra finch

**Category**	**TE**	**Benchmark**	**EDTA**^**a**^	**EDTA_repbase**^**b**^	**EDTA_repeatmodeler2**^**c**^	**RepeatModeler2**^**d**^
Animals	LTR	Sensitivity	63.3%	57.6%	72.2%	81.4%
Animals	non-LTR	Sensitivity	1.0%	43.7%	66.9%	76.1%
Animals	TIR	Sensitivity	38.8%	35.1%	40.9%	38.9%
Plants	LTR	Sensitivity	94.7%	94.6%	94.7%	73.5%
Plants	non-LTR	Sensitivity	0.0%	82.2%	49.6%	57.5%
Plants	TIR	Sensitivity	67.1%	66.8%	67.1%	28.1%
Animals	LTR	Specificity	95.0%	97.8%	96.9%	96.8%
Animals	non-LTR	Specificity	100.0%	98.2%	98.7%	97.9%
Animals	TIR	Specificity	88.4%	91.7%	89.8%	97.4%
Plants	LTR	Specificity	90.4%	90.7%	90.5%	97.9%
Plants	non-LTR	Specificity	100.0%	99.6%	99.9%	99.9%
Plants	TIR	Specificity	94.7%	95.1%	94.8%	99.6%

^a^EDTA run with the --sensitive 1 parameter

^b^EDTA run with the --curatedlib repbase-nonLTR.fasta input

^c^EDTA run with the --curatedlib RepeatModeler2-nonLTR.fasta input

^d^RepeatModeler2 run with default parameters

EDTA has been under constant development since its original release, with many of the improvements originating from user feedback as detailed on the EDTA GitHub repository. The commentary by Gozashti and Hoekstra provides further guidance for improvement. We appreciate the points raised in the commentary on the generalized use of EDTA. We are currently developing a new version of EDTA that, among other improvements, will contain a non-LTR module using RepeatModeler2 [4] in conjunction with TEsorter, and potentially other programs such as AnnoSINE [5], that will wrap the execution of non-LTR retrotransposon annotations directly into the EDTA framework. Even with these species-agnostic improvements, we cannot emphasize enough the importance of incorporating known information about the specific TE content of an organism into the annotation process to maximize the performance of any TE annotation software. In the case of EDTA, this is most simply done through the incorporation of a reference TE library that includes known species-specific TE sequence information.

Beyond the incorporation of tools that have been developed and improved since the original release of EDTA 1.0 in 2019, we also see the need for improvements to the underlying TE annotation algorithms for a number of different types of TEs. The tools for annotation of non-LTR retrotransposons are still underdeveloped relative to LTR retrotransposons. There is a need to develop tools specifically for the structural annotation of LINEs, a major subclass of non-LTR retrotransposons, rather than relying on homology-based approaches. Improvement of automated Helitron annotation algorithms will also have a profound impact as there is currently a very high false positive rate during the annotation of Helitrons that also contributes to misclassification of other types of TEs. As new tools become available, we will continue to benchmark them and incorporate those that improve the overall performance of EDTA. We sincerely hope the entire TE community, particularly those who study non-plant genomes, join our effort to develop tools for annotation of genomes with diverse TE landscapes.

## Data Availability

Not applicable.
